# Longitudinal associations between BMI, ideal-actual BMI gap, and body shape concern among young Chinese females

**DOI:** 10.3389/fpubh.2025.1549695

**Published:** 2025-05-08

**Authors:** Yebo Yu, Siyan Yuan, Jie Wang, Xuemei Zhao, Lutong Li, Hewei Min, Siyu Dong, Dongxia Yu, Xinying Sun

**Affiliations:** Department of Social Medicine and Health Education, School of Public Health, Peking University, Beijing, China

**Keywords:** body shape concern, ideal-actual BMI gap, young females, cross-lagged panel model, non-linear associations

## Abstract

**Objectives:**

Body shape concerns significantly impact young females' psychological wellbeing. This study aimed to estimate the short-term bidirectional relationships among BMI, ideal-actual BMI gap, and body shape concern across different BMI groups, and further explore their potential non-linear associations in young Chinese females.

**Methods:**

We conducted a longitudinal study among Chinese females aged 18 to 30 in December 2023 (T1) and April 2024 (T2). Body mass index (BMI) was calculated using the formula: weight in kilograms divided by the square of height in meters, based on self-reported data. The body shape questionnaire 8-item version C (BSQ-8C) was adopted to measure levels of body shape concern. We utilized two-time-point cross-lagged panel models (CLPMs) to investigate temporal associations among BMI, ideal-actual BMI gap, and body shape concern, and used restricted cubic spline (RCS) fitted for multiple linear regressions to explore their potential non-linear relationships.

**Results:**

A total of 688 young females were enrolled (mean age = 21.084, SD = 2.091). The percentages of underweight, normal, and overweight-obesity were 12.2%, 66.9%, and 20.9%, respectively. In the normal and overweight-obesity groups, the ideal BMI was significantly lower than the actual BMI at baseline. Among underweight females, 44.70% expressed a desire to further reduce their BMI. For all participants, the higher the BMI at T1, the smaller the ideal-actual BMI gap at T2, which means the more the ideal value of BMI was lower than its actual value at T2. In the normal BMI group, the ideal-actual BMI gap and body shape concern negatively predicted each other. A U-shaped correlation was observed between baseline body shape concern and BMI change in the overweight group.

**Conclusion:**

Complex reciprocal effects of BMI, ideal-actual BMI gap, and body shape concern existed in different BMI groups. There is an urgent need for the whole society to pay more attention to the issue of body shape concern. In particular, health educators should organize programs to promote accurate weight perception among young women, and policymakers should enhance content regulation by restricting the promotion of extreme weight loss across media platforms. This approach would help avoid the negative impact of excessive concerns about body image on mental health.

## 1 Introduction

The subject of body weight has always been a focal point of interest within the domain of public health. According to the World Health Organization, in 2022, ~43% of the global adult population was classified as overweight, and 16% as obese (1). Concurrently, the prevalence of underweight individuals was noted to be 7.6% among females and 6.8% among males (2). In China, the rates of overweight (37.5%), obesity (8.7%), and underweight (4.8%) were lower than the global average in 2022. Nonetheless, given China's substantial population, an estimated 48 million males and 37 million females aged 18–69 years were obese in 2022, potentially contributing to a significant disease burden (3). It is indicated that the overweight rate in China is projected to rise to 65.3% by 2030, with related healthcare expenditures accounting for 21.5% of the total cost (4). Both underweight and overweight statuses are associated with an increased risk of adverse health outcomes throughout the lifespan (5).

It is reported that discrepancies between perceived and actual weight are evident, with men more likely to underestimate their BMI and women to overestimate it (6, 7). Moreover, the tendency to overestimate weight reaches its highest level in the 20–29 age group among women (8). A European study across seven countries found that 20% of women with a BMI of 20 kg/m^2^ viewed themselves as “a bit overweight” or “too fat,” and the percentage rose to 60% for those with a BMI of 22.5 kg/m^2^ (9). Japanese and Korean women's ideal weight and BMI are significantly below current levels (10, 11). In China, 47.0% of girls considered themselves overweight (12). This discrepancy between actual and ideal weight may serve as a predictor of future weight fluctuations. A 15-year longitudinal study found that among individuals aiming to lose weight, underweight males gained significant weight, while those with a BMI above 25 kg/m^2^ lost more weight (7). Regrettably, there is limited literature exploring the gap between ideal and actual body weight and examining its impact on weight changes, particularly within the context of China.

The gap between ideal and actual weight may stem from sociocultural impacts. Influenced by a confluence of traditional culture, social customs, and mass media (13), body weight has emerged as a paramount objective measure of physical attractiveness (14). A cross-national study demonstrated an inverse linear relationship between the physical attractiveness of females and their body mass index (BMI) in all ethnic groups (15), suggesting the pervasiveness of the sociocultural ideal of “thinness as beauty.” A study conducted in China found that perceived sociocultural pressure contributed to thin-ideal internalization and body shame, and the conceptualization of beauty may lead to anti-fat attitudes, such as disliking fat people (16). In western countries, a very slim physique has been promoted by the media for decades (17) and remains dominant aesthetic standard for women (18). However, some scholars argue that western culture trends have recently shifted toward a curvier body type named “slim-thick,” characterized by full hips and thighs, a slim waist, and a flat abdomen (17, 19).

Amid this societal and cultural emphasis on slenderness, an increasing number of individuals express dissatisfaction or anxiety regarding their weight, driven not by health concerns but by aesthetic expectations shaped by social interactions (20). This negative attitude toward body image is prevalent among young women, with 30.6% reporting concerns about their body shape and 7.3% indicating significant to moderate concerns in India (21). These figures are even more pronounced in China, where 87.4% of young women are dissatisfied with their bodies, and 72.8% want to be slimmer (22). Such body shape concerns may lead individuals to adopt unhealthy weight control practices (23) and even result in more serious psychological problems (24). Consequently, addressing body shape concerns among young females is an urgent priority that requires focused attention and intervention. Unhealthy body weight and unhealthy quests for weight loss are likely to worsen concerns or dissatisfaction with body image. It is suggested that BMI is positively linked to body dissatisfaction (25) and body shape concern (26). Additionally, existing studies indicate that a larger discrepancy between ideal and actual weight, as well as between ideal body shape and perceived body shape, is associated with lower body shape satisfaction (27). Weight and body image are key components of an individual's self-identity and self-esteem (28). A substantial discrepancy between the ideal and actual weight may lead to lower self-esteem, thereby increasing body shape concerns. However, there is a lack of longitudinal studies exploring how weight and weight disparities affect body image concerns, making it difficult to establish causal relationships between these variables.

Young women are in a stage of both physical development and psychological maturation, where their body image perception is highly susceptible to the influence of social and cultural factors. It is indicated that healthy weight management behaviors adopted during early adulthood significantly shape health habits in later adulthood, potentially having a greater impact than behaviors during adolescence (29). Understanding the current situation of the weight gap between reality and ideals as well as body shape concern, and examining their links to BMI changes among young women across different BMI categories, is crucial for promoting healthy weight perspectives and encouraging rational weight management behaviors. But in fact, body image concerns among young Chinese women remain poorly understood, and the dynamic associations between weight change, ideal-actual weight gap, and body image concerns are still unclear. To address this, we conducted a 4-month longitudinal study focusing on young Chinese females, aiming to (1) track changes in BMI, ideal-actual BMI gap, and body image concerns, (2) investigate the short-term bidirectional relationships between these three variables at baseline (T1) and month 4 (T2) using cross-lagged panel models (CLPMs), (3) and explore potential non-linear associations among study variables by applying restricted cubic spline (RCS) analysis in different BMI groups. The study's findings are expected to clarify how young women perceive weight in the context of current Chinese sociocultural factors, provide evidence for health intervention strategies targeting women at different BMI levels, and offer valuable insights for the formulation of future public health policies related to health education and weight management.

## 2 Materials and methods

### 2.1 Study design

This 4-month longitudinal study focused on young Chinese females and was carried out from December 2023 to April 2024. Cluster sampling and convenience sampling methods were used to recruit participants. Recruitment information was posted on popular Chinese new media platforms, including Bilibili, Weibo, Xiaohongshu, and Douyin (30). These platforms each have over 30% of their user base composed of young women aged 18 to 30, though their user groups differ in characteristics (31): Bilibili users are often described as young, individualistic, and culturally engaged, with a strong focus on content aesthetics; Douyin users are diverse, covering a wide range of age groups; Xiaohongshu users are primarily young women with relatively higher education and income levels; and Weibo users, while also predominantly young, exhibit greater variability in education levels. Collectively, these platforms are estimated to reach 585 million to 665 million young female users, making them a reasonably representative sample of China's young female population (31). We then adopted convenience sampling to select study participants from volunteers who signed up for this research. Data collection was conducted through online surveys to ensure participant anonymity, using a professional platform named “SurveyStar.” All participants were informed about the study's purpose, anonymity, confidentiality, and other rights. Informed consent was obtained from all participants before their voluntary involvement in the research.

After posting the recruitment information, our team received a total of 1,830 enrollment emails and successfully contacted 1,334 young women, of whom 819 completed the online survey at baseline (T1). After excluding 50 male respondents and 17 participants who were either under 18 or over 30 years old, 752 eligible participants were included in this study. At the 4-month follow-up (T2), 688 participants completed the second online survey. Since there was no significant difference in sociodemographic characteristics between the 752 baseline participants and the 688 follow-up participants (see Supplementary Table S1), we focused our analysis on the 688 participants, aged 18 to 30, who completed the online surveys two times. The study was approved by the Biomedical Ethics Committee of Peking University in China (IRB00001052-22076).

### 2.2 Measurement

#### 2.2.1 Body shape concern

The Body Shape Questionnaire (BSQ), which consists of 34 questions, was initially developed by Cooper et al. (32) to assess body shape dissatisfaction caused by feeling fat. Pook evaluated several short forms of the BSQ and found that BSQ-8C (BSQ-8 items-version C) showed a reasonable fit and high sensitivity (33), which was first proposed by Evans and Dolan (34). BSQ-8C was believed to have high test-retest reliability, internal consistency, and convergent validity (35). There are 8 items in BSQ-8C, responding on a 6-point Likert-like scale from “never” to “always.” The total score is the sum of the scores of all the items. The higher the total score, the higher the level of the body shape concern. The Cronbach's alpha was 0.914 at T1 and 0.910 at T2 in this study.

#### 2.2.2 BMI, ideal-actual BMI gap, and BMI change

Body mass index (BMI) was calculated using self-reported weight and height, applying the formula of weight in kilograms divided by the square of height in meters. Previous studies have shown that the differences between self-reported and actual weight and height are minimal (36), particularly among adolescents and young adults (37, 38). Moreover, web-based self-reported weight is considered a method with high validity and reliability (39). In this study, participants were young females who were highly conscious of their body image. It is likely that most of them regularly monitor their weight (40), which means they were likely aware of their weight status when completing the questionnaire. Therefore, the self-reported height and weight data collected in this study were deemed accurate and valid. Based on the guideline for Chinese individuals, BMI was divided into four categories: underweight (BMI < 18.5), normal (18.5 ≤ BMI < 24.0), overweight (24.0 ≤ BMI < 28.0), and obesity (BMI ≥ 28.0) (41). In this study, we merged overweight and obesity into a single group, referred to as the “overweight-obesity” group.

In addition to actual weight, participants were asked about their ideal weight at T1 and T2, from which ideal BMI was calculated. The ideal-actual BMI gap was defined as the difference between ideal BMI and actual BMI (calculated as ideal BMI minus actual BMI). Finally, BMI change was calculated by subtracting the actual BMI at T1 from the actual BMI at T2 (calculated as BMI at T2 minus BMI at T1).

#### 2.2.3 Socio-demographic factors

Socio-demographic variables included age, nationality (Han/else), residence (urban/rural), the highest education level (high school and below/undergraduates and above), and monthly household income per capita (≤ 3,000/3,001-5,000/≥5,001, yuan/RMB).

### 2.3 Statistical analysis

Data analyses were performed using SPSS version 26.0 and R program 4.2.1. Descriptive analysis was used to describe the basic characteristics of the participants using frequency (percentage) and mean (standard deviation). The chi-square test and one-way ANOVA were used to compare characteristics among different BMI groups. Then, we adopted a paired sample *t*-test to compare body weight and BMI at different time points (T1 and T2) and to test the difference between actual and ideal BMI at T1.

A cross-lagged panel model (CLPM) was performed using the R package “lavaan” to investigate temporal associations among BMI, ideal-actual BMI gap, and body shape concern. The CLPM is capable of examining autoregressive effects (the association of the same variable across different time points), cross-lagged effects (the predictive influence of one variable on another variable at a subsequent time point), and contemporaneous effects (the correlations between different variables at the same time point) (42). The Cross-Lagged Panel Model (CLPM) assumes measurement invariance, meaning that the measurement of the same variable must remain consistent across different time points, as well as stationarity, referring to consistent autoregressive and cross-lagged effects across different time intervals. This study conducted linear regressions to assess the lagged effects, using two-time point measurements of BMI, ideal-actual BMI gap, and the scores of body shape concern. The Akaike Information Criterion (AIC), the Bayesian Information Criterion (BIC), Comparative Fit Index (CFI), Goodness of Fit Index (GFI), and Standardized Root Mean Square Residual (SRMR) were provided to present the relative goodness of fit (43). The CLPM including all paths has zero degrees of freedom and is a saturated model, making it impossible to obtain model fit indices (44). To obtain reasonable models and optimal model fit indices, we conducted *post hoc* modifications by removing the path that was non-significant in the results of all BMI groups (T1 Body shape concern → T2 BMI) (45). Furthermore, CLPM was conducted among the underweight group, the normal group, and the overweight-obesity group, respectively. And we also conducted CLPM in the overweight group and obesity group separately. We used the “semPower” package in R to calculate the *post hoc* power (46). With the number of observed variables being 6 and α = 0.05, we input each model fit index (GFI) and degrees of freedom, and obtained the values of *post hoc* power for each BMI group.

Then, we used restricted cubic spline (RCS) fitted for multiple linear regression to explore the potential non-linear and dose-response relationships between BMI change with T1 ideal-actual BMI gap and T1 body shape concern and between T2 body shape concern and T1 ideal-actual BMI gap. R package “rms” was used to establish RCS models among all participants and each BMI groups, respectively. All models were adjusted for age, nationality, residency, the highest education level, and monthly household income per capita. In the spline models, three to five knots were suggested, considering the smoothness of the curve and avoiding the loss of accuracy caused by overfitting (47). We compared the goodness of fit of each model with 3, 4, or 5 knots and selected the number of knots with the minimum AIC value (48). Statistical significance was considered as *P* < 0.05. Besides, adopting G^*^power software (49), we calculated effect size *f*^2^ and corresponding *post hoc* powers for all participants and each BMI group, using *R*^2^ of each RCS model, the number of observed variables = 6, and α = 0.05.

## 3 Results

### 3.1 Background information and weight change

Table 1 shows that the mean age of all participants was 21.084 ± 2.091. Among 688 female participants, the percentages of underweight, normal, and overweight-obesity people were 12.2%, 66.9%, and 20.9%, respectively. There were differences in the scores of body shape concern at T1 and T2 among different BMI groups (*P* < 0.001).

**Table 1:** The sociodemographic characteristics at T1 and body shape concern at T1 and T2 of participants (*N* = 688).

**Characteristics**	**Total**	**Underweight**	**Normal**	**Overweight-obesity**	***F*/*χ^2^***	***P* value**
	***N*** **(%)**	***N*** **(%)**	***N*** **(%)**	***N*** **(%)**		
Female	688 (100)	84 (12.2)	460 (66.9)	144 (20.9)		
Age (mean ± SD)	21.084 ± 2.091	21.060 ± 2.014	20.996 ± 2.069	21.382 ± 2.193	1.882	0.153
**Nationality**					1.398	0.497
Han	643 (93.5)	76 (90.5)	432 (93.9)	135 (93.8)		
Else	45 (6.5)	8 (9.5)	28 (6.1)	9 (6.3)		
**Residency**					0.284	0.868
Rural	35 (5.1)	5 (6)	22 (4.8)	8 (5.6)		
Urban	653 (94.9)	79 (94)	438 (95.2)	136 (94.4)		
**The highest education level**					1.931	0.381
High school and below	38 (5.5)	3 (3.6)	24 (5.2)	11 (7.6)		
Undergraduates and above	650 (94.5)	81 (96.4)	436 (94.8)	133 (92.4)		
**Monthly household income per capita (RMB)**					1.204	0.877
≤ 3,000	239 (34.7)	29 (34.5)	161 (35)	49 (34)		
3,001–5,000	201 (29.2)	26 (31)	137 (29.8)	38 (26.4)		
≥5,001	248 (36)	29 (34.5)	162 (35.2)	57 (39.6)		
**Scores of body shape concern**						
Score at T1 (mean ± SD)	22.789 ± 9.561	16.512 ± 7.523	22.196 ± 9.053	28.347 ± 9.374	49.432	< 0.001
Score at T2 (mean ± SD)	22.458 ± 8.704	17.810 ± 7.936	21.926 ± 8.352	26.868 ± 8.388	34.360	< 0.001

T1, baseline. T2, Month 4. In order to compare characteristics among different groups, one-way ANOVA was used for age and scores of body shape concern, and the Chi-square test was used for other variables.

**Figure 1:**
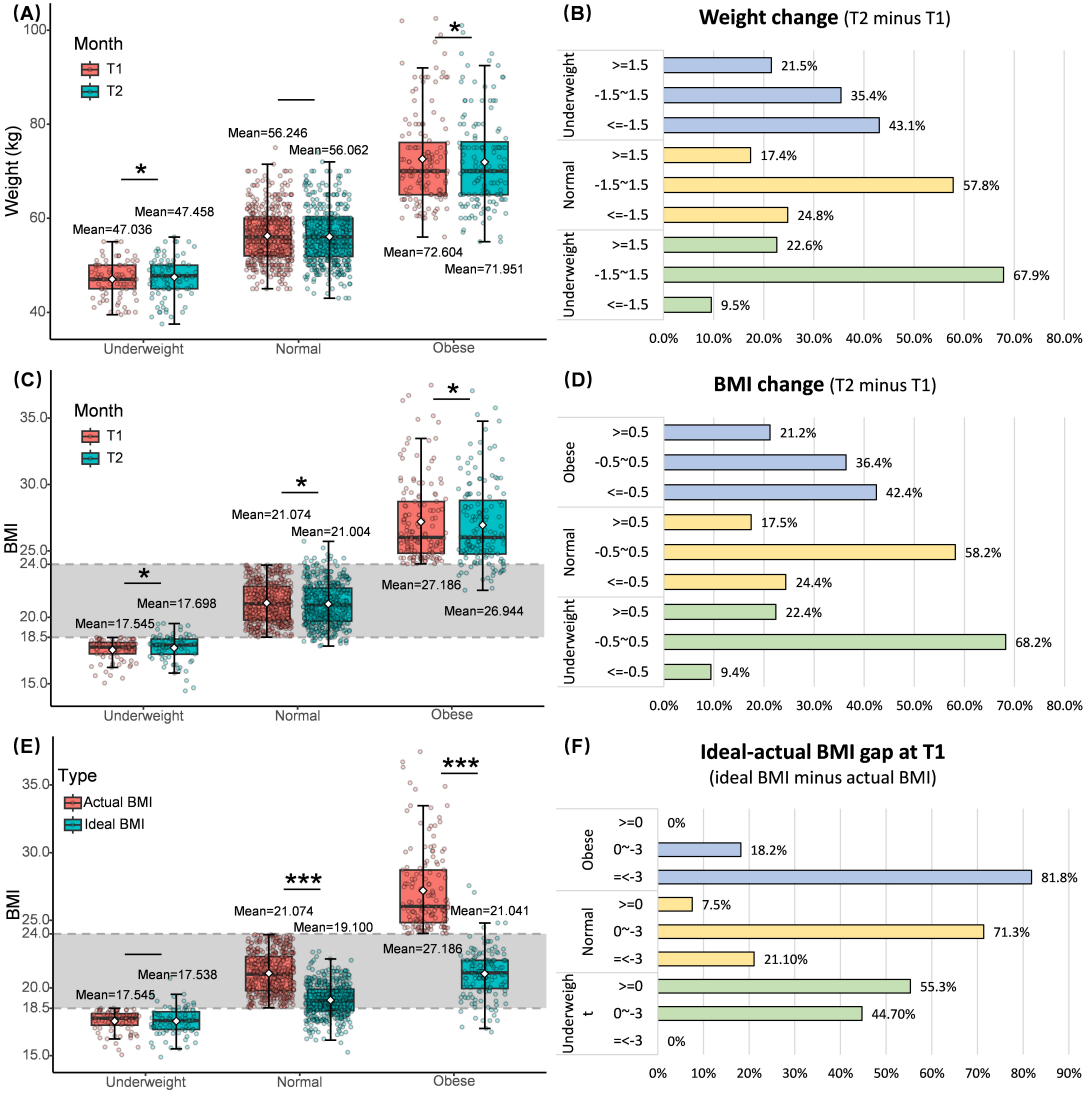
The comparison of body weight **(A)** and BMI **(C)** at T1 and T2 and their changes **(B, D)**, and the difference **(E)** and gap **(F)** between actual and ideal BMI at T1. T1, baseline. T2, Month 4. **(A, C, E)** Paired sample *t*-test was used to examine the difference between T1 and T2 for each BMI group, ***P < 0.001, **P < 0.01, *P < 0.05. The white point inside the box plot represents the mean value of the corresponding group.

The body weight and BMI increased significantly from T1 to T2 in participants of the underweight group. However, 9.5% of underweight participants lost weight by more than 1.5 kg. Participants whose weight was in the normal range at T1 had no significant change in weight and a significant reduction in BMI from T1 to T2. In the overweight-obesity group, weight and BMI had a significant drop from T1 to T2, above 40% of them decreased BMI by more than 0.5 kg/m^2^ (see Figures 1A–D). The ideal BMI is significantly lower than the actual BMI for participants in the normal and overweight-obesity groups at T1. In addition, 44.3% of participants in the underweight group at T1 wanted to reduce their BMI by 0 to 3 units (see Figures 1E, F).

### 3.2 Cross-lagged panel model

**Figure 2:**
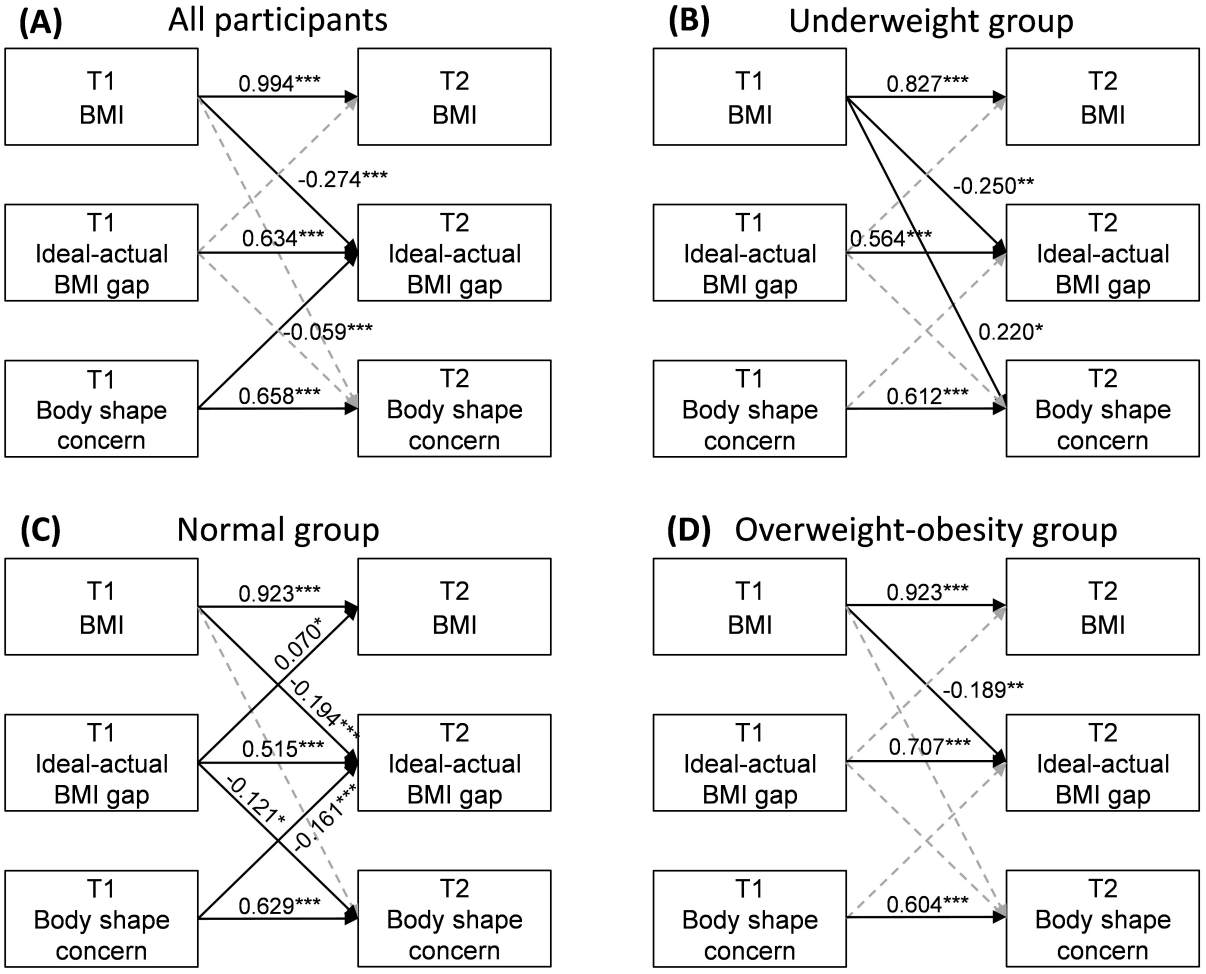
Standardized regression coefficients in cross-lagged panel model in all participants **(A)**, underweight group **(B)**, normal group **(C)**, and overweight-obesity group **(D)**. T1, baseline. T2, Month 4. Ideal-actual BMI gap: ideal BMI at T1 minus actual BMI at T1. ***P < 0.001, **P < 0.01, *P < 0.05. Only paths of cross-lagged effects and autoregressive effects were presented in the above figure. The detailed results of CLPMs are shown in Supplementary Table S3. Solid lines present significant paths, and dashed lines present non-significant paths.

Figure 2 presents the cross-lagged panel models of BMI, ideal-actual BMI gap, and body shape concern at T1 and T2 for all participants and different BMI groups. The autoregressive effects of BMI, ideal-actual BMI gap, and body shape concern were significant in all models (*P* < 0.001). Regardless of the baseline BMI, the higher the BMI at baseline, the smaller the ideal-actual BMI gap at T2, that is, the more the ideal weight is lower than the actual weight (*P* < 0.05). Meanwhile, the BMI of the underweight group at T1 was positively associated with their scores of body shape concern at T2 (β = 0.220, *P* = 0.010). In the normal group, the ideal-actual BMI gap at T1 could predict the BMI (β = 0.070, *P* = 0.016) and body shape concern (β = −0.121, *P* = 0.011) at T2, and body shape concern at T1 had a negative relationship with ideal-actual BMI gap at T2 (β = −0.161, *P* < 0.001). The detailed path coefficients and model fit indices are shown in Supplementary Table S2.

Then we conducted CLPMs for the overweight and obesity groups, respectively. In the overweight group, T1 BMI (β = −0.209, *P* = 0.012) and T1 body shape concern (β = −0.142, *P* = 0.016) were negatively associated with T2 ideal-actual BMI gap. While for the obesity group, only autoregressive paths were significant (see Supplementary Table S3). The *post hoc* powers of CLPMs are shown in Supplementary Table S4.

### 3.3 Non-linear associations

**Figure 3:**
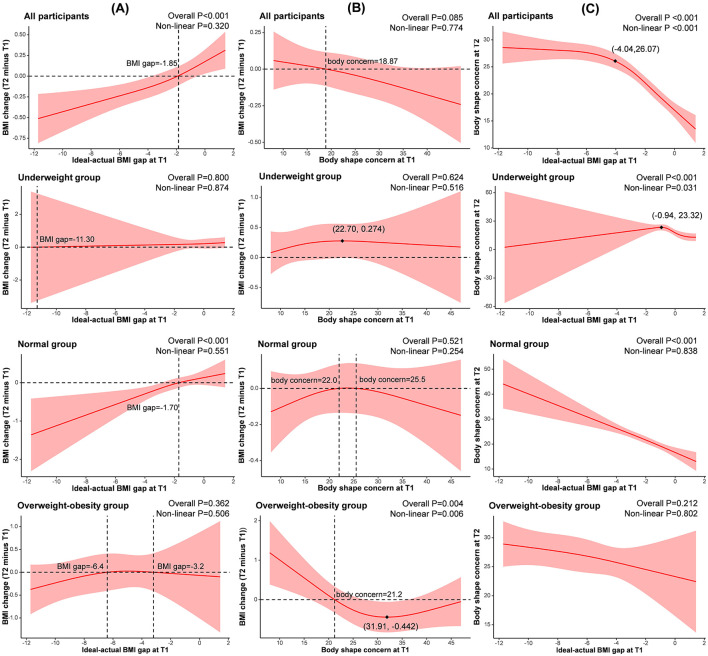
The non-linear associations between **(A)** BMI change with T1 ideal-actual BMI gap, **(B)** BMI change with T1 body shape concern, and **(C)** T2 body shape concern with T1 ideal-actual BMI gap. T1, baseline. T2, Month 4. BMI change, BMI at T2 minus BMI at T1. Ideal-actual BMI gap, ideal BMI at T1 minus actual BMI at T1. All models above were adjusted by age, nationality, residency, the highest education level, and monthly household income per capita.

The AIC values produced during the process of model selection are presented in Supplementary Table S5. As shown in Figure 3A, BMI change (T2 minus T1) had a positive linear association with the ideal-actual BMI gap at T1 in the normal group (β = 0.281, *P* < 0.001). In the overweight-obesity group, there was a U-shaped association between BMI change and T1 body shape concern (non-linear *P* = 0.006), reaching the lowest point at (31.91, −0.442; see Figure 3B). Figure 3C presents the association between T2 body shape concern and T1 ideal-actual BMI gap, which was non-linear in the underweight group (non-linear *P* = 0.031), and negatively linear in the normal group (β = −6.020, *P* < 0.001). In the overweight group, T1 body shape concern was U-shaped associated with BMI change (non-linear *P* = 0.014; Supplementary Figure S1B). The *post hoc* powers of RCS models are shown in Supplementary Table S6.

## 4 Discussion

Our study enrolled and followed up with 688 Chinese women aged 18–30 years to investigate the complex relationships among BMI, ideal-actual BMI gap, and body shape concern. At baseline, we observed that the ideal BMI was significantly lower than the actual BMI in both the normal and overweight-obesity groups. After a 4-month follow-up period, BMI increased significantly in the underweight group and decreased significantly in the normal and overweight-obesity groups. In all BMI groups, BMI at T1 negatively predicted the ideal-actual BMI gap at T2. Within the normal BMI group, significant negative bidirectional relationships were identified between the ideal-actual BMI gap and body shape concern. In the underweight group, BMI at T1 positively predicted body shape concern at T2. Additionally, a U-shaped correlation was observed between baseline body shape concern and BMI change in the overweight group.

Ideal weight or BMI reflects the accuracy of perceptions regarding a healthy weight. As our findings demonstrated, both normal and overweight-obesity groups had significantly lower ideal BMIs compared to their actual BMI, and their ideal BMI remained within the healthy range. It is suggested that overweight and obese women are aware of the health risks associated with obesity, which is consistent with previous research (50). Young women with normal BMI expressed a desire to achieve a lower BMI, potentially due to an overestimation of their weight, under the premise of maintaining health (51). Additionally, nearly half of the underweight females aspired to further reduce their BMI by 0–3 units, potentially worsening their underweight status and increasing the risk of malnutrition. One contributing factor to this discrepancy in weight perceptions may be the difference in learning and active engagement with health-related knowledge between the underweight group and the normal and overweight-obesity groups (52). Under the pressure of social aesthetics, women who are obese or even those with a normal weight may actively seek information on weight management, leading to a more comprehensive understanding of weight health and fostering more accurate weight attitudes compared to their underweight counterparts (53). Besides, underweight females, influenced by societal aesthetics that emphasize thinness, may prioritize appearance and exhibit a stronger inclination toward extreme slimness (54). The results of our subsequent qualitative interviews further corroborate these findings. We randomly selected two subjects from those who were underweight and whose ideal weight was lower than their actual weight. The basic information of interviewees is provided in Supplementary Table S7, and the interview outline is shown in Supplementary Table S8. During the interviews, both participants expressed distinct stereotypes about individuals with thin and obese body types. They associated thin people with traits such as “efficient,” “better at getting things done,” “more disciplined,” and “clean.” In contrast, they perceived obese people as “unclean,” “lacking self-control,” and “likely to have poorer work performance.” One interviewee also mentioned that she felt “better-looking and more attractive” when her weight was lower than her actual weight. And she attributed this perception to “sociocultural influences,” stating that “everyone seems to think that being thinner is more attractive.” Thus, under the combined influence of sociocultural pressures and insufficient health knowledge, young women who are already underweight aspire to enhance their appeal and improve their social image by pursuing an even slimmer figure (55, 56).

Across all BMI groups, we found that BMI at T1 was negatively associated with the T2 ideal-actual BMI gap. This suggests that individuals with higher baseline BMIs tend to exhibit a smaller ideal-actual BMI gap, reflecting a stronger motivation to reduce weight. Our finding is consistent with studies conducted in Finland (57) and America (58). Furthermore, we identified that only in the normal BMI group did the ideal-actual BMI gap at T1 positively predict BMI at T2, and the T1 ideal-actual BMI gap was positively associated with BMI change. This indicates that a stronger desire to lose weight in individuals with a normal BMI is associated with a subsequent reduction in BMI. Individuals with a normal BMI are more likely to maintain a positive attitude toward weight management and tend to take healthy and reasonable measures to reduce weight (59), resulting in a decrease in BMI within the normal range (60). In contrast, an ideal weight that significantly deviates from actual weight may prompt some overweight and obese people to adopt unhealthy or inappropriate weight loss methods (7, 25), which may contribute to the failure of weight loss. For underweight females, weight reduction is neither necessary nor easy when starting from a baseline weight below the healthy threshold. However, the underweight group had a small sample size, which may reduce the statistical power of the cross-lagged panel model, potentially leading to false-negative results regarding the influence of the ideal-actual BMI gap on BMI (61).

At baseline, scores of body shape concern increased sequentially from the underweight to the overweight-obesity groups, consistent with prior research indicating a positive correlation between higher BMI and greater body shape concern (62, 63). In addition, through subgroup analyses stratified by BMI, we examined the longitudinal associations between BMI and body image concerns. Notably, the results revealed that the positive relationship between baseline BMI and future body shape concern was only significant in the underweight group, but not in the normal and overweight-obesity groups. Slim females tend to be sensitive to perceived weight changes and are more likely to overestimate their weight (64), potentially resulting in a subsequent rise in body image concerns (65).

Inversely, BMI change was also influenced by the level of baseline body shape concern. A U-shaped association was observed between BMI change and baseline body shape concern in overweight individuals. Specifically, BMI decreased when body shape concern scores ranged from 23.3 to 42.2; BMI increased when body shape concern scores were >42.2 or < 23.3. However, this U-shaped association was not significant in the obesity group. First, the lack of significance in the obesity group may be partially attributed to its lower statistical power. Second, on a psychological level, overweight females may have higher expectations of their body shape compared to the obese population (66). Moderate levels of body shape concern may serve as a motivational factor for adopting positive and healthy weight management strategies, thereby facilitating successful weight loss. Whereas, excessive anxiety about body shape may induce overweight women to adopt unhealthy ways of losing weight, such as extreme dieting or the development of eating disorder symptoms, including binge eating, which may result in unsuccessful weight loss or rapid weight regain (67). In contrast, individuals with obesity may have experienced prolonged exposure to an obese state, leading to psychological adaptation to their body weight. Their level of body shape concern may not sufficiently motivate immediate or significant weight reduction actions (67, 68). It is plausible that excessive concern about body shape prompts obese women to adopt rational weight-loss methods. Therefore, in the short term (few months), the effect of body shape concern on BMI for obese females is not as obvious as that of overweight individuals. Third, among the overweight and obesity population, restrictive dieting behaviors have negative impacts on both weight loss effectiveness and mental health (69), while regular exercise behaviors have positive effects. Hence, dieting and exercise may influence the association between BMI changes and body shape concern. Unfortunately, these confounding factors were not measured in this study. Fourth, from a biological perspective, leptin, a hormone involved in appetite regulation and metabolic processes, exhibits a strong correlation with BMI (70). In overweight individuals, excessively high levels of body shape concern may disrupt the secretion or function of leptin and other weight-regulating hormones (71), thereby impairing metabolic homeostasis and potentially contributing to an increase in BMI. While the metabolic dysregulation observed in individuals with obesity may be more resistant to modification and less influenced by body shape concern. It is also possible that obese individuals have more complex metabolic and hormonal regulatory mechanisms that differ from those in the overweight population (72). As a result, excessive body shape concern in overweight individuals not only poses risks to mental health (24) but also may compromise weight loss efforts due to associated psychological distress (73). It is crucial to recognize that when body shape concern scores exceed a specific threshold, their reducing effect on BMI becomes ineffective, and BMI may continue to increase in overweight females.

Moreover, we investigated the bidirectional associations between the ideal-actual BMI gap and body shape concern. In the normal BMI group, the ideal-actual BMI gap and body shape concern negatively affect each other. Unrealistic aspirations for a lower weight may drive individuals within the normal BMI range to pursue thinness excessively, potentially neglecting their psychological wellbeing. According to self-discrepancy theory (74), a large disparity between the actual self and the ideal self may induce cognitive dissonance in body image perception, thereby exacerbating anxiety and body image concerns. Additionally, self-esteem or body image satisfaction may play a mediating factor in the pathway linking the ideal-actual BMI gap to body shape concern (75). Furthermore, some young women might adopt inappropriate weight loss behaviors (76), which may lead to a slow and deleterious weight loss process, and which may further undermine their body confidence (20, 77). Conversely, a significant negative impact of body shape concern on the ideal-actual BMI gap was observed exclusively among women with normal weight. This finding can be attributed to the fact that the ideal weight perceptions of both obese and underweight individuals are primarily influenced by health-related considerations, rather than body shape concerns.

Several limitations of this study should be acknowledged. First, convenience sampling was adopted and participation in the surveys was voluntary. This approach may introduce selection bias, thereby limiting the generalizability of the sample and the applicability of the findings. Second, the weight and body shape concerns in the study were collected using self-reported questionnaires, which may be susceptible to reporting bias. Third, despite the inclusion of certain socio-demographic variables such as age, ethnicity, and educational attainment in the analysis, this study did not account for other factors associated with body image anxiety such as self-esteem (78), media exposure (28), and social anxiety (79). The absence of adjustments for these confounders may lead to an overestimation of the effect of BMI change on body shape concern (80). The study also did not measure dietary-related factors and physical activity, neglecting their impacts on weight changes and mental health, which potentially reduce the accuracy of the correlational results. Fourth, the follow-up period in this study was limited to 4 months, which may be insufficient to capture the long-term changes in BMI and their impact on mental health. Nevertheless, some studies indicated that young females' BMI may fluctuate significantly over short periods (81). Weight change influenced by weight attitude (82) and the interactive effects between body shape concern and weight changes (83, 84) could also be observed in short-term follow-ups. In this study, short-term associations between BMI, ideal-actual BMI gap, and body shape concerns were identified. Further research is necessary to explore their long-term relationships. Fifth, when subgroup analyses of the CLPMs and the RCS models were conducted, some BMI groups had smaller sample sizes, which may have reduced statistical power and hindered the detection of actual effects (61). Consequently, negative results in groups with small sample sizes should be interpreted with caution. Finally, in this study, we conducted subgroup analyses stratified by BMI to identify differences among BMI groups and validate the robustness of the findings. Future studies should include formal sensitivity analyses to further confirm the robustness of the results.

Despite the aforementioned limitations, this study explored the dynamic relationships between BMI, ideal-actual BMI gap, and body shape concern among young women, offering significant theoretical and practical value. First of all, a substantial proportion of young women in the underweight group have an ideal weight that is lower than their actual weight. The pronounced ideal-actual BMI discrepancy highlights the pervasive influence of the societal norm equating thinness with beauty among young women, serving as a primary driver of body-related anxiety. The finding underscores the importance of enhancing health education targeting young women to promote accurate weight perception, and to prevent physical and mental health disorders such as eating disorders and malnutrition as a result of extreme thinner pursuits. Furthermore, the large discrepancy between ideal and actual body weight might exacerbate body shape concerns among women of normal weight. It is necessary to be vigilant about the boundary between healthy weight loss and pathological weight loss. A reduction in BMI accompanied by excessive body shape concern may indicate an elevated risk of developing eating disorders. Therefore, we cannot ignore females with normal BMI in health education activities, as they may be at risk of mental health problems due to their unrealistic weight goals. Finally, as body shape concern is exacerbated, BMI change in the overweight group exhibits an initial decline followed by an increase. This trend suggests that encouraging overweight women to maintain a reasonable level of body shape concern could be an effective strategy to facilitate weight loss. It is imperative to monitor the intensity of body shape concern, as excessive body shape concern may be closely associated with weight gain or other severe psychological issues. Therefore, the level of body shape concern should be regarded as a significant indicator in the practical implementation of weight loss interventions for overweight individuals.

This article also highlights several potential applications that warrant emphasis. First, health education initiatives should be implemented in universities and communities to assist young women in developing accurate health awareness, reducing their reliance on a singular aesthetic standard, and mitigating concerns related to body image. For individuals exhibiting elevated levels of body shape concern, cognitive-behavioral therapy (CBT) may be utilized to alleviate body-related anxiety and prevent extreme weight-loss behaviors. Second, young women with varying baseline BMI levels should adopt tailored weight management strategies. For instance, individuals classified as overweight or obese may benefit from aerobic exercise to reduce body fat, while those who are underweight may engage in resistance training to increase muscle mass. Third, it is recommended that media platforms enhance content regulation by incorporating warning labels on materials promoting extreme weight loss and increasing the visibility of body diversity content through algorithmic adjustments. Such measures can help counteract the societal norm that equates thinness with beauty. Fourth, the findings suggest that future public health policies should prioritize the promotion of accurate weight perception and weight management skills among young women, fostering the development of a healthy body image from an early age.

## 5 Conclusion

In summary, this study revealed the longitudinal associations among BMI, ideal-actual BMI gap, and body shape concern using CLPM and RCS. We discovered that BMI at T1 positively predicted body shape concern at T2 for underweight females. In the overweight group, a U-shaped correlation was observed between baseline body shape concern and BMI change. Additionally, the normal BMI group had bidirectional relationships between ideal-actual BMI gap and body shape concern. Our findings highlight that the overall weight perception level of young Chinese women needs to be improved. In health education activities, underweight women should be encouraged to increase muscle mass and pursue a healthy beauty, while normal-weight women should be helped to alleviate body shape concerns and enhance body satisfaction. The adverse effects of body shape concern on weight loss outcomes and psychological wellbeing should be taken into account when designing weight loss strategies for the overweight population.

## Data Availability

The datasets presented in this article are not readily available because this is an ongoing research project, and data applications will be allowed at the end of the project (2030). Requests to access the datasets should be directed to https://sph.pku.edu.cn/info/1544/3938.htm.
