# Supplementary material for: Longitudinal associations between BMI, ideal-actual BMI gap, and body shape concern among young Chinese females
Source: Front Public Health. 2025 May 8;13:1549695. doi: 10.3389/fpubh.2025.1549695 (PMC12094990; doi:10.3389/fpubh.2025.1549695)
Supplement: Supplementary file 1 [file Data_Sheet_1.pdf]

Supplementary materials

Table S1. The comparison of baseline characteristics between 752 participants at T1 and 688 participants at T2 who completed surveys twice.

	Participants at T1	Participants at T2	F/χ^2	*P* value
	N (%)	N (%)		
Female	752 (100)	688 (100)		
Age (mean±SD)	21.088±2.111	21.084±2.091	0.031	0.975
Nationality			<0.001	0.985
Han	703 (93.5)	643 (93.5)		
Else	49 (6.5)	45 (6.5)		
Residency			0.279	0.597
Rural	709 (94.3)	35 (5.1)		
Urban	43 (5.7)	653 (94.9)		
The highest education level			0.140	0.708
High school and below	45 (6.0)	38 (5.5)		
Undergraduates and above	707 (94.0)	650 (94.5)		
Monthly household income per capita (RMB)			0.226	0.893
≤3000	255 (33.9)	239 (34.7)		
3001-5000	217 (28.9)	201 (29.2)		
≥5001	280 (37.2)	248 (36)		
BMI group			0.086	0.958
Thin	90 (12.0)	93 (13.5)		
Normal	508 (67.6)	462 (67.2)		
Overweight or obesity	154 (20.5)	133 (19.3)		
Scores of body shape concern	22.786±9.592	22.789±9.561	0.007	0.995

Note. To compare characteristics among different groups, one-way ANOVA was used for age and scores of body shape concern, and the Chi-square test was used for other variables.

Table S2. The detailed results of cross-lagged panel models among BMI, ideal-actual BMI gap, and body shape concern.

	All participants			Underweight group			Normal BMI group			Overweight-obesity group		
	β	SE	*P*-value	*β*	SE	*P*-value	*β*	SE	*P*-value	*β*	SE	*P*-value
**Regressions:**												
T1 BMI→T2 BMI	0.994	0.020	<0.001	0.827	0.098	<0.001	0.923	0.030	<0.001	0.923	0.056	<0.001
T1 BMI→T2 ideal-actual BMI gap	-0.274	0.025	<0.001	-0.250	0.137	0.004	-0.194	0.036	<0.001	-0.189	0.069	0.008
T1 BMI→T2 body shape concern	0.018	0.147	0.755	0.220	0.846	0.010	-0.014	0.246	0.752	0.080	0.328	0.500
T1 ideal-actual BMI gap→T2 BMI	0.028	0.026	0.169	0.064	0.067	0.404	0.070	0.035	0.016	-0.005	0.057	0.928
T1 ideal-actual BMI gap→T2 ideal-actual BMI gap	0.634	0.033	<0.001	0.564	0.097	<0.001	0.515	0.044	<0.001	0.707	0.070	<0.001
T1 ideal-actual BMI gap→T2 body shape concern	-0.075	0.202	0.221	-0.091	0.602	0.306	-0.121	0.304	0.011	0.062	0.338	0.606
T1 body shape concern→T2 ideal-actual BMI gap	-0.059	0.004	<0.001	-0.022	0.012	0.766	-0.161	0.004	<0.001	-0.021	0.011	0.541
T1 body shape concern→T2 body shape concern	0.658	0.027	<0.001	0.612	0.081	<0.001	0.629	0.034	<0.001	0.604	0.062	<0.001
**Covariances:**												
T1 BMI→T1 ideal-actual BMI gap	-0.888	0.465	<0.001	-0.518	0.114	<0.001	-0.642	0.108	<0.001	-0.824	0.974	<0.001
T1 BMI→T1 body shape concern	0.389	1.360	<0.001	0.210	0.663	0.059	0.234	0.649	<0.001	0.144	2.373	0.087
T1 ideal-actual BMI gap→T1 body shape concern	-0.456	1.049	<0.001	-0.344	1.003	0.003	-0.422	0.593	<0.001	-0.238	2.390	0.006
T2 BMI→T2 ideal-actual BMI gap	-0.569	0.038	<0.001	-0.385	0.061	0.001	-0.587	0.036	<0.001	-0.572	0.155	<0.001
T2 BMI→T2 body shape concern	0.128	0.199	0.001	0.119	0.352	0.279	0.182	0.214	<0.001	0.050	0.641	0.552
T2 ideal-actual BMI gap→T2 body shape concern	-0.184	0.247	<0.001	-0.172	0.500	0.121	-0.285	0.261	<0.001	-0.037	0.783	0.656
**Variances:**												
T1 BMI	1.000	0.653	<0.001	1.000	0.098	<0.001	1.000	0.148	<0.001	1.000	1.073	<0.001
T1 ideal-actual BMI gap	1.000	0.370	<0.001	1.000	0.208	<0.001	1.000	0.111	<0.001	1.000	1.052	<0.001
T1 body shape concern	1.000	4.921	<0.001	1.000	8.628	<0.001	1.000	5.392	<0.001	1.000	10.283	<0.001
T2 BMI	0.060	0.038	<0.001	0.367	0.057	<0.001	0.227	0.037	<0.001	0.140	0.155	<0.001
T2 ideal-actual BMI gap	0.162	0.057	<0.001	0.462	0.113	<0.001	0.458	0.052	<0.001	0.235	0.232	<0.001
T2 body shape concern	0.504	2.058	<0.001	0.452	4.286	<0.001	0.532	2.410	<0.001	0.637	5.284	<0.001

**Goodness of fit**				
χ^2	0.005	1.860	3.425	3.599
df	1.000	1.000	1.000	1.000
AIC	18682.477	1911.374	11219.531	4222.429
BIC	18773.153	1959.991	11302.156	4281.825
CFI	1.000	0.997	0.999	0.997
GFI	1.000	0.993	0.998	0.992
SRMR	<0.001	0.024	0.013	0.018

Note. T1: baseline. T2: Month 4. β: standardized coefficient. SE: standardized error. Ideal-actual BMI gap: ideal BMI minus actual BMI.

Table S3. The cross-lagged panel models of BMI, ideal-actual BMI gap, and body shape concern among overweight and obese participants.

	Overweight group			Obesity group		
	β	SE	*P*-value	*β*	SE	*P*-value
**Regressions:**						
T1 BMI→T2 BMI	0.713	0.126	<0.001	0.889	0.086	<0.001
T1 BMI→T2 ideal-actual BMI gap	-0.209	0.146	0.012	-0.101	0.112	0.342
T1 BMI→T2 body shape concern	0.059	0.817	0.549	0.027	0.363	0.825
T1 ideal-actual BMI gap→T2 BMI	0.006	0.078	0.938	0.010	0.083	0.910
T1 ideal-actual BMI gap→T2 ideal-actual BMI gap	0.511	0.093	<0.001	0.770	0.109	<0.001
T1 ideal-actual BMI gap→T2 body shape concern	0.074	0.533	0.479	-0.035	0.355	0.771
T1 body shape concern→T2 ideal-actual BMI gap	-0.142	0.011	0.016	0.111	0.022	0.133
T1 body shape concern→T2 body shape concern	0.556	0.083	<0.001	0.751	0.081	<0.001
**Covariances:**						
T1 BMI→T1 ideal-actual BMI gap	-0.518	0.206	<0.001	-0.616	1.122	<0.001
T1 BMI→T1 body shape concern	0.218	1.044	0.035	0.133	3.326	0.371
T1 ideal-actual BMI gap→T1 body shape concern	-0.367	1.748	0.001	-0.159	3.428	0.288
T2 BMI→T2 ideal-actual BMI gap	-0.640	0.177	<0.001	-0.491	0.284	0.003
T2 BMI→T2 body shape concern	0.116	0.843	0.254	-0.144	0.833	0.333
T2 ideal-actual BMI gap→T2 body shape concern	-0.154	0.982	0.131	0.249	1.111	0.102
**Variances:**						
T1 BMI	1.000	0.161	<0.001	1.000	1.316	<0.001
T1 ideal-actual BMI gap	1.000	0.417	<0.001	1.000	1.388	<0.001
T1 body shape concern	1.000	12.919	<0.001	1.000	16.515	<0.001
T2 BMI	0.497	0.182	<0.001	0.220	0.276	<0.001
T2 ideal-actual BMI gap	0.498	0.245	<0.001	0.320	0.471	<0.001
T2 body shape concern	0.702	7.698	<0.001	0.419	4.933	<0.001
**Goodness of fit**						
χ^2		3.505			0.889	
df		1.000			1.000	
AIC		2634.937			1353.290	
BIC		2686.637			1389.863	
CFI		0.990			1.000	
GFI		0.988			0.994	
SRMR		0.038			0.021	

Note. T1: baseline. T2: Month 4. β: standardized coefficient. SE: standardized error. Ideal-actual BMI gap: ideal BMI minus actual BMI.

Table S4. The post hoc powers of cross-lagged panel models (CLPMs) for each BMI group.

BMI group	N (%)	Power
All participants	688 (100)	/
Thin	84 (12.2)	0.263
Normal	460 (66.9)	0.383
Overweight and obesity	144 (20.9)	0.460
Overweight	98 (14.2)	0.468
Obesity	46 (6.7)	0.069

Note. The CLMP of all participants has an SRMR close to 0, making it impossible to calculate its post hoc power.

Table S5. AIC values of non-linear association models with different knots.

		AIC			Knots
		3 knots	4 knots	5 knots	selected
All participants	Model 1	**1724.735**	1726.736	1728.479	3 knots
	Model2	**1746.246**	1747.980	1749.711	3 knots
	Model 3	4798.753	**4797.761**	4799.740	4 knots
Thin	Model 1	**170.734**	172.717	174.689	3 knots
	Model2	**170.179**	172.152	174.141	3 knots
	Model 3	580.282	**574.803**	576.498	4 knots
Normal	Model 1	**1053.110**	1054.123	1055.861	3 knots
	Model2	**1068.753**	1070.391	1072.293	3 knots
	Model 3	**3205.576**	3207.439	3208.843	3 knots
Overweight and obesity	Model 1	**464.299**	466.040	465.385	3 knots
	Model2	**454.896**	455.765	457.668	3 knots
	Model 3	**1032.965**	1034.872	1035.433	3 knots
Overweight	Model 1	317.015	**314.619**	315.420	4 knots
	Model2	**308.631**	310.062	311.849	3 knots
	Model 3	**709.066**	709.854	710.668	3 knots
Obesity	Model 1	158.307	**155.988**	157.957	4 knots
	Model2	**159.910**	160.760	162.540	3 knots
	Model 3	331.578	**326.348**	328.589	4 knots

Note. Model 1: Y=BMI change, X=T1 ideal-actual BMI gap; Model 2: Y=BMI change, X=T1 body shape concern; Model 3: Y=T2 body shape concern, X=T1 ideal-actual BMI. All models above were adjusted by age, nationality, residency, the highest education level, and monthly household income per capita.

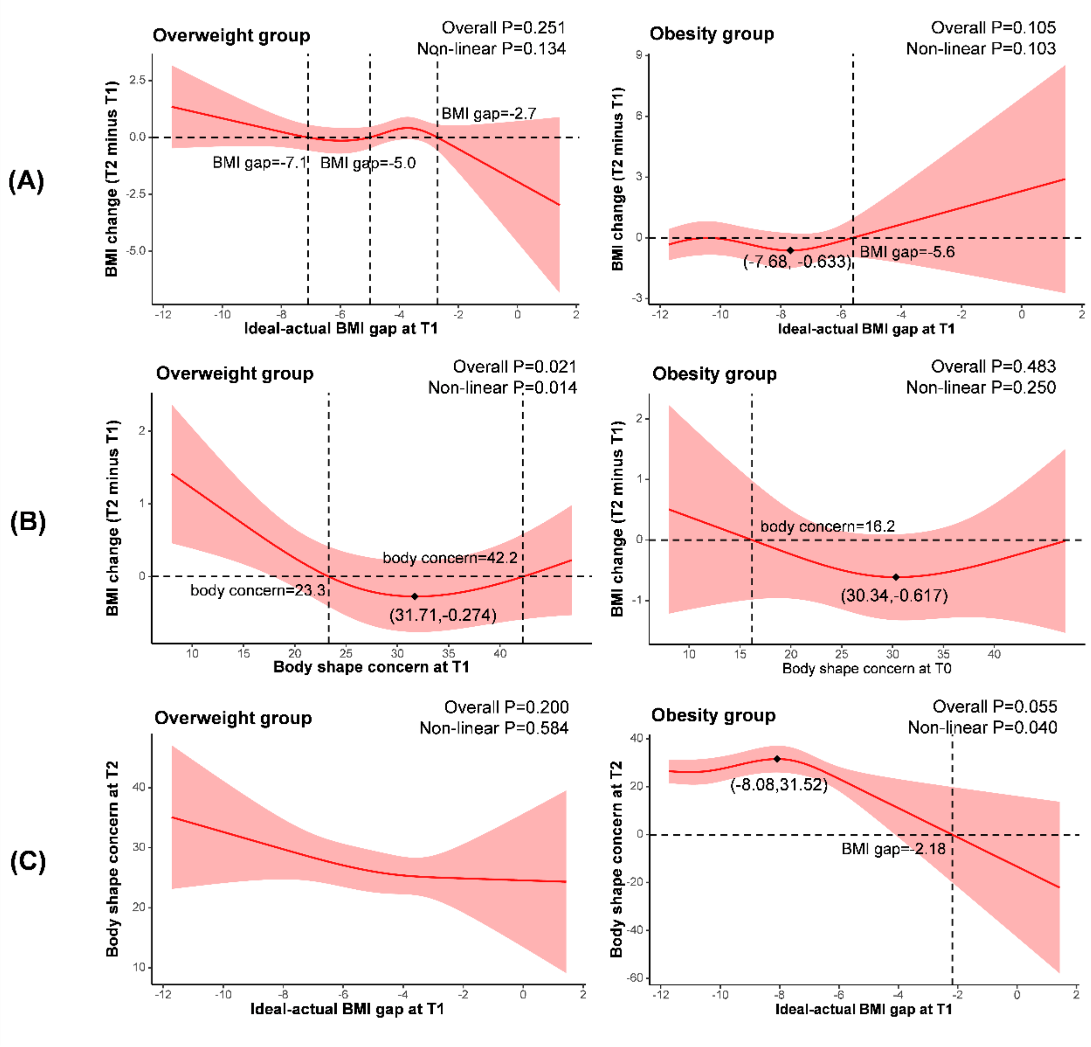

Figure S1. The non-linear associations for overweight and obesity groups between (A) BMI change with T1 ideal-actual BMI gap, (B) BMI change with T1 body shape concern, and (C) T2 body shape concern with T1 ideal-actual BMI gap.

Note. T1: baseline. T2: Month 4. BMI change: BMI at T2 minus BMI at T1. Ideal-actual BMI gap: ideal BMI at T1 minus actual BMI at T1. All models above were adjusted by age, nationality, residency, the highest education level, and monthly household income per capita.

Table S6. The post hoc power of restricted cubic spline (RCS) models for each BMI group.

		R^2	Effect size f^2	Power
	Model 1	0.041	0.043	0.993
All participants	Model 2	0.010	0.010	0.456
	Model 3	0.200	0.250	1.000
	Model 1	0.055	0.058	0.303
Thin	Model 2	0.061	0.065	0.339
	Model 3	0.321	0.473	0.999
	Model 1	0.046	0.048	0.959
Normal	Model 2	0.013	0.013	0.396
	Model 3	0.144	0.168	1.000
	Model 1	0.054	0.057	0.521
Overweight and obesity	Model 2	0.132	0.152	0.951
	Model 3	0.121	0.138	0.927
	Model 1	0.096	0.106	0.628
Overweight	Model 2	0.132	0.152	0.810
	Model 3	0.121	0.138	0.764
	Model 1	0.253	0.339	0.789
Obesity	Model 2	0.150	0.176	0.464
	Model 3	0.240	0.316	0.755

Note. Model 1: Y=BMI change, X=T1 ideal-actual BMI gap; Model 2: Y=BMI change, X=T1 body shape concern; Model 3: Y=T2 body shape concern, X=T1 ideal-actual BMI. All models above were adjusted by age, nationality, residency, the highest education level, and monthly household income per capita. Effect size $f^2 = R^2/(1-R^2)$.

Table S7. Sociodemographic information of interviewees

	Interviewee 1	Interviewee 2
Age	20	19
Gender	Female	Female
Nationality	Han	Han
Residency	Urban	Urban
The highest education level	Undergraduates	Undergraduates
Monthly household income per capita (RMB)	⩾5001	⩾5001
Height (m)	1.62	1.70
Weight at T1 (kg)	48.5	50.0
Ideal weight at T1 (kg)	45.0	45.0

Table S8. The brief outline of the interview

No.	Quention
Quention 1	Please use a few words to describe your impression of an overweight or obese person.
Quention 2	Please use a few words to describe your impression of someone who is underweight.
Quention 3	Your weight is already below the normal range, why do you want to lose weight further?
Quention 4	How do your family and friends perceive your weight? What kind of weight management advice do they give you?
Quention 5	Who is your favorite celebrity? To what extent is your body preference influenced by him/her?
Quention 6	Does being slim give you some advantages or hindrances in your social life? Why?
Quention 7	How would it affect your health if you lost weight to your goal? Will it influence your mental state?
